# GapmeR cellular internalization by macropinocytosis induces sequence-specific gene silencing in human primary T-cells

**DOI:** 10.1038/srep37721

**Published:** 2016-11-24

**Authors:** Mobashar Hussain Urf Turabe Fazil, Seow Theng Ong, Madhavi Latha Somaraju Chalasani, Jian Hui Low, Atish Kizhakeyil, Akshay Mamidi, Carey Fang Hui Lim, Graham D. Wright, Rajamani Lakshminarayanan, Dermot Kelleher, Navin Kumar Verma

**Affiliations:** 1 Lymphocyte Signalling Research Laboratory, Lee Kong Chian School of Medicine, Nanyang Technological University, 636921, Singapore; 2 School of Chemical and Biomedical Engineering, Nanyang Technological University, 637459, Singapore; 3 School of Biological Sciences, Nanyang Technological University, 637551, Singapore; 4 Institute of Medical Biology, A*STAR, Immunos, Biopolis, 138648, Singapore; 5 Singapore Eye Research Institute, The Academia, 20 College Road, 169856, Singapore; 6 Faculty of Medicine, University of British Columbia, Vancouver, British Columbia V1Y 1T3, Canada

## Abstract

Post-transcriptional gene silencing holds great promise in discovery research for addressing intricate biological questions and as therapeutics. While various gene silencing approaches, such as siRNA and CRISPR-Cas9 techniques, are available, these cannot be effectively applied to “hard-to-transfect” primary T-lymphocytes. The locked nucleic acid-conjugated chimeric antisense oligonucleotide, called “GapmeR”, is an emerging new class of gene silencing molecule. Here, we show that GapmeR internalizes into human primary T-cells through macropinocytosis. Internalized GapmeR molecules can associate with SNX5-positive macropinosomes in T-cells, as detected by super-resolution microscopy. Utilizing the intrinsic self-internalizing capability of GapmeR, we demonstrate significant and specific depletion (>70%) of the expression of 5 different endogenous proteins with varying molecular weights (18 kDa Stathmin, 80 kDa PKCε, 180 kDa CD11a, 220 kDa Talin1 and 450 kDa CG-NAP/AKAP450) in human primary and cultured T-cells. Further functional analysis confirms CG-NAP and Stathmin as regulators of T-cell motility. Thus, in addition to screening, identifying or verifying critical roles of various proteins in T-cell functioning, this study provides novel opportunities to silence individual or multiple genes in a subset of purified human primary T-cells that would be exploited as future therapeutics.

T-lymphocytes are the principal effector cells of the adaptive immune system. To better understand the biology of T-cells in health and their role in chronic inflammation, autoimmunity and lymphoid cancers, it becomes imperative to perform specific knockdown of target genes in primary T-cells under various experimental conditions. In addition, specific modulation of T-cell functions by silencing genes of interest in purified T-cell subsets has emerged as an attractive approach to augment immunity for cancer adoptive cellular therapies^1^. However, dissection of many intracellular signalling pathways involved in the regulation of human T-cell functions and development of gene silencing-based immunotherapeutics have been hampered due to problems associated with delivering of inhibitory constructs.

The RNA interference (RNAi) and CRISPR-Cas9 techniques are being increasingly used for targeted gene silencing in a diverse range of primary and cultured mammalian cells in the laboratory settings. However, the exploitation of these tools for post-transcriptional gene silencing in biological/translational research or as therapeutics aimed at targeting T-cells has been hampered by the fact that lymphocytes are conventionally “hard-to-transfect”^2^,^3^, *i.e.* they are resistant to transfection reagents (*e.g.* cationic lipids and polymers) and they also possibly lack an efficient RNAi machinery^4^. Although antisense molecules or small interfering RNAs (siRNAs) can be transduced into T-cells by electroporation or nucleofection *in vitro*^5^,^6^, the high electrical pulses that are applied to transiently permeablize the cell membrane in these methods typically result in significant loss of cell viability. Other gene silencing techniques that have been applied in T-cells include viral-, nanoparticle- and aptamer-based interfering RNA delivery systems and Accell-modified siRNAs^7^,^8^; but these methods yield low transfection efficiency. In addition, siRNAs have poor stability, could have off-target effects^9^, and may trigger unwanted immune responses^10^,^11^.

The locked nucleic acid (LNA)-conjugated chimeric single-strand antisense oligonucleotide, called “GapmeR”, is an emerging new class of molecule, which can knockdown a target gene of interest with precise specificity through the post-transcriptional gene silencing^12^. Typically, a GapmeR molecule contains 2–5 chemically modified LNA as “wings” at each terminus flanking a central “gap” of 5–10 base single strand antisense DNA. The combination of both chemistry and structural modifications provides GapmeR with high binding affinity for the target mRNA, confers resistance to nucleases imparting improved stability in biological serum and cell culture medium, and facilitates unassisted cellular uptake or “gymnosis”^13^. Notably, addition of 3 LNA molecules increases GapmeR half-life up to 10-times^14^ and use of shorter length oligos minimizes the possibility of an immunogenic response^13^. Once a GapmeR molecule enters the cell, the central antisense DNA in the gap binds to the endogenous mRNA resulting in a GapmeR-mRNA duplex. The GapmeR-mRNA duplex is recognized by the cellular enzyme RNase H, which degrades the targeted mRNA and thus inhibits specific gene expression^12^,^13^. The sequence of the DNA gap and flanking LNAs can be carefully designed using available gene sequence database and bioinformatics tools to achieve high target affinity, sequence specificity, biological stability, and favourable pharmacokinetic and tissue-penetrating properties.

There are multiple mechanisms by which small oligonucleotides (*e.g.* interfering RNAs) or their cationic complexes can internalize into mammalian cells. These include phagocytosis, pinocytosis, clathrin- and caveolin-dependent endocytosis. In particular, a type of endocytosis called “macropinocytosis” mediates non-selective uptake of tiny molecules, such as viruses, bacteria, nanoparticles, nutrients and antigens^15^. Macropinocytosis is initiated from cell surface membrane ruffles that fold back onto themselves forming heterogeneous-sized endocytic structures known as macropinosomes^15^. Fluid-phase substances get trapped in macropinosomes and are then delivered into the cytoplasm. A member of the sorting nexin family of proteins, SNX5, has been found to be associated with macropinosomes^16^.

Herein, we show that GapmeR molecules can interact with intracellular SNX5-vesicles and internalize into T-cells through a macropinocytosis-like endocytic mechanism *i.e.* in the absence of transfection reagents or electroporation. Specifically designed GapmeR could silence target genes of interest in human primary T-cells with precise specificity and high efficiency.

## Results

### GapmeR molecules are self-internalized by primary human T-cells

**Figure 1:**
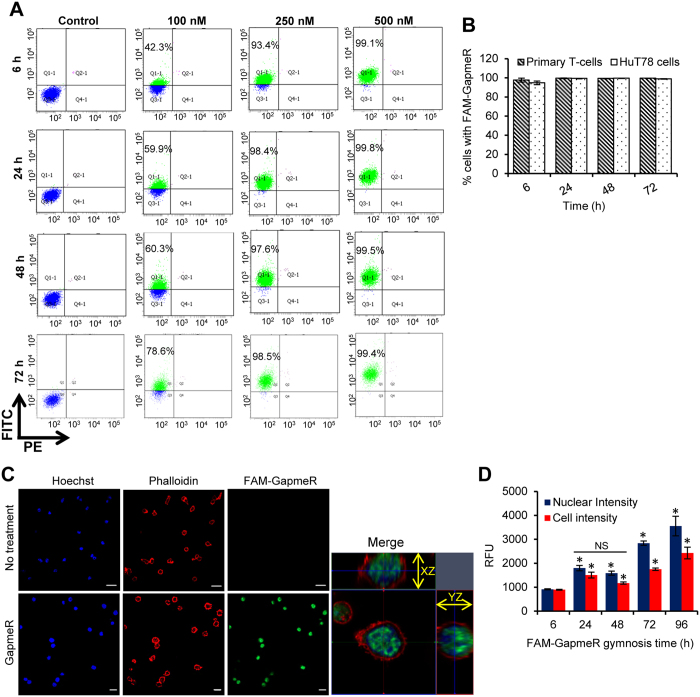
Cellular internalization of GapmeR delivered through direct uptake in human T-cells. (**A**) Primary human T-cells were incubated with 100 nM, 250 nM or 500 nM non-targeting FAM-GapmeR for 6 h, 24 h, 48 h or 72 h and then GapmeR cellular uptake was analysed by flow cytometry. Results show dose- and time-dependent cellular internalization of GapmeR. (**B**) Both primary human T-cells and HuT78 cells were incubated separately with 500 nM FAM-GapmeR for up to 72 h and GapmeR cellular uptake was analysed by flow cytometry. Results (mean ± SEM) showed comparable rate of GapmeR internalization in HuT78 and primary T-cells. (**C**) Primary T-cells were incubated with 500 nM FAM-GapmeR (green) for 6 h, fixed and counter-stained with Rhodamine-Phalloidin (to visualize cells, red) and Hoechst (to visualized nuclei, blue). GapmeR cellular localization was analysed by confocal microscopy (63X oil objective lens). Images clearly show cytoplasmic and nuclear localization of GapmeR. (**D**) Time-dependent cellular internalization of FAM-GapmeR delivered through gymnosis (*cell intensity* and *nuclear intensity*) was quantified using High Content Analysis. Data (mean ± SEM) represent at least three independent experiments using T-cells purified from at least 3 different donors. Differences in nuclear/cell intensities between 24 h and 48 h treatments were non-significant (*NS*); *p < 0.05.

Initially, we incubated human primary T-cells with various concentrations of FAM-labelled non-targeting GapmeR (100 nM, 250 nM or 500 nM) for various time points (6 h, 24 h, 48 h or 72 h). At the end of treatment periods, GapmeR cellular uptake was analysed using flow-cytometery. Data clearly showed dose-dependent cellular internalization of GapmeR through direct uptake “gymnosis” and ~60% T-cells were transfected with 100 nM FAM-GapmeR in 24 h (Fig. 1A). At 500 nM concentration, FAM-GapmeR showed close to 100% transfection efficiency even at 6 h that sustained for up to 72 h (Fig. 1A). Similar results on cellular uptake of FAM-GapmeR were obtained in HuT78 T-cells incubated with various concentrations of FAM-GapmeR ranging from 10 nM to 500 nM (gymnotic delivery) or transfected through nucleofection (Supplementary Fig. S1). Comparable amount of GapmeR cellular uptake through gymnosis was evident in both primary human T-cells and HuT78 cells following incubation with 500 nM FAM-GapmeR for various time-points ranging from 6 to 72 h (Fig. 1B). To further detect cellular internalization of GapmeR in T-cells, we performed confocal, super-resolution and 3D Structured Illumination Microscopy (3D-SIM) of FAM-GapmeR treated T-cells. Confocal microscopic images of primary T-cells or HuT78 cells incubated with 500 nM FAM-GapmeR for 6 h or 48 h showed GapmeR localization in the cytoplasm as well as in the nucleus (Fig. 1C, Supplementary Fig. S2, Supplementary Movie 1). Super-resolution and 3D-SIM microscopy of HuT78 T-cells treated with 500 nM FAM-GapmeR molecules further confirmed their cellular targeting (Supplementary Movies 2, 3a and 3b). Interestingly, internalized GapmeR molecules displayed “doughnut-shaped” vesicular-like structures within the cell (Supplementary Movies 2 and 3a). High Content Analysis of primary T-cells and HuT78 cells showed time-dependent increase in the internalization of GapmeR in both cytoplasm as well as nucleus (Fig. 1D, Supplementary Fig. S2D). Similar results on the cellular uptake of FAM-GapmeR were obtained with other cell-types, including primary human dermal fibroblasts, lung epithelial carcinoma cell line A549 and hepatocellular carcinoma cell line HepG2, as visualized by confocal microscopy (Supplementary Fig. S3).

### GapmeR molecules do not interfere with cell viability and are non-immunogenic

**Figure 2:**
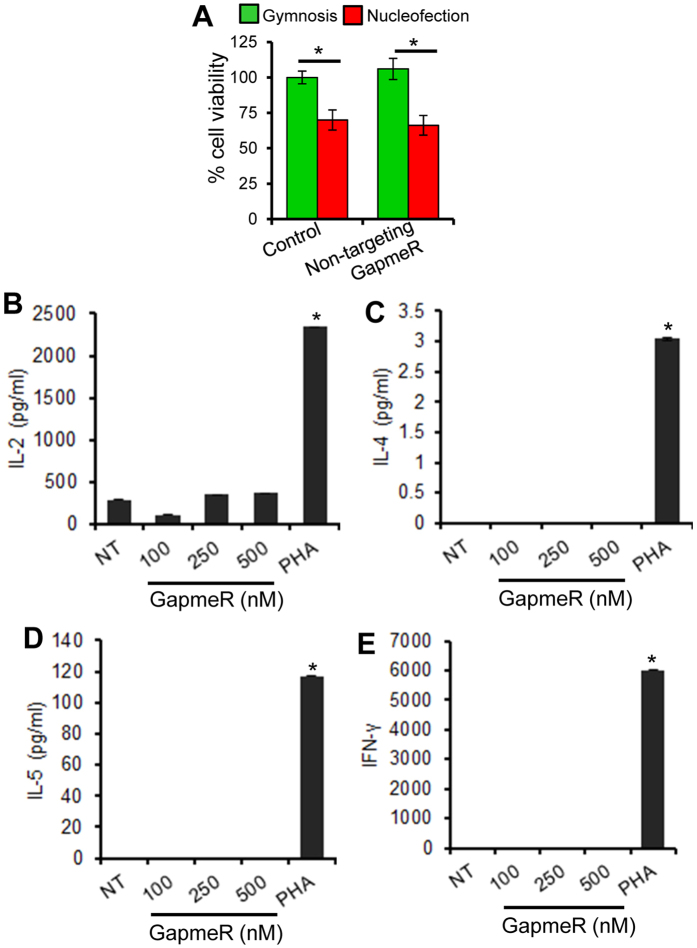
Effect of GapmeR treatment on T-cell viability and immunogenic responses. (**A**) Human primary T-cells were incubated with 1 μM non-targeting GapmeR to allow gymnosis or transfected by nucleofection. Equivalent amount of PBS was used as control. After 48 h, percentage cell viability was determined and presented. (**B–E**) Human primary T-cells were incubated with 100, 250 or 500 nM non-targeting GapmeR for 24 h. Cells were left untreated (*NT*) or treated with phytohemagglutinin (*PHA*) as negative and positive controls. After centrifugation, supernatant culture media were collected and secreted levels of cytokines IL-2 (**B**), IL-4 (**C**), IL-5 (**D**) and IFN-γ (**E**) were analysed by ELISA. Data (mean ± SEM) represent three independent experiments using T-cells purified from at least 3 different donors, *p < 0.05.

There was no appreciable loss in cell viability due to the incubation of T-cells with as high as 1 μM non-targeting GapmeR for 24 h (Fig. 2A, green bars); however, nucleofection procedure alone caused significant loss in cell viability (Fig. 2A, red bars). Moreover, GapmeR treatment did not induce any unwanted immunogenic response in human primary T-cells *i.e.* there was no detectable secretion of IL-2, IL-4, IL-5 or IFN-γ due to non-targeting GapmeR (Fig. 2B–E).

### GapmeR internalizes to T-cells through macropinocytosis mechanism

**Figure 3:**
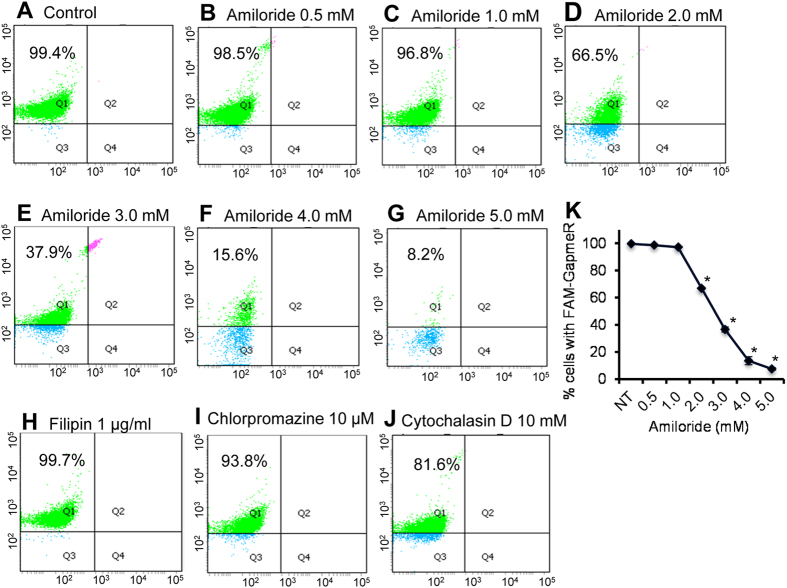
Effect of endocytosis inhibitors on GapmeR cellular internalization in human T-cells. Primary human T-cells were untreated (**A**, *control*) or pre-treated with amiloride [0.5 mM (**B**), 1.0 mM (**C**), 2.0 mM (**D**), 3.0 mM (**E**), 4.0 mM (**F**) or 5.0 mM (**G**)], 1 μg/ml filipin (**H**), 10 μM chlorpromazine (**I**), 10 mM cytochalasin D (**J**) for 30 min. Cells were then incubated with 500 nM FAM-GapmeR for 24 h to allow gymnosis. Cellular internalization of GapmeR was analysed by flow cytometry. Results show dose-dependent inhibition of FAM-GapmeR cellular uptake in cells treated with amiloride (**K**) but not with other three inhibitors. Data represent at least three independent experiments using T-cells purified from at least 3 different donors, *p < 0.05.

There are many possible ways by which small molecules, such as GapmeR, could be internalized into mammalian cells. These include pinocytosis, phagocytosis, clathrin-mediated endocytosis or caveolae-mediated uptake process^15^. To investigate the mechanism of GapmeR cellular uptake in primary human T-cells, we treated cells separately with specific endocytosis pathway inhibitors, including amiloride (pinocytosis inhibitor^17^), cytochalasin D (phagocytosis inhibitor^18^), filipin (caveolae-mediated uptake inhibitor^19^), or chlorpromazine (clathrin-mediated endocytosis inhibitor^19^,^20^). Pre-treatment of T-cells with increasing concentrations of amiloride (0.5 to 5 mM), prior to incubation with FAM-GapmeR, showed dose-dependent inhibition of GapmeR internalization, with >95% inhibition at 5 mM concentration (Fig. 3A–G, K). However, presence of inhibitory concentrations of filipin (1 μg/ml), chlorpromazine (1 μM), or cytochalasin D (10 mM) had no significant effect on GapmeR cellular uptake (Fig. 3H–J). Please note that the higher concentrations of filipin, chlorpromazine, or cytochalasin D than indicated above were found to be cytotoxic (data not shown).

**Figure 4:**
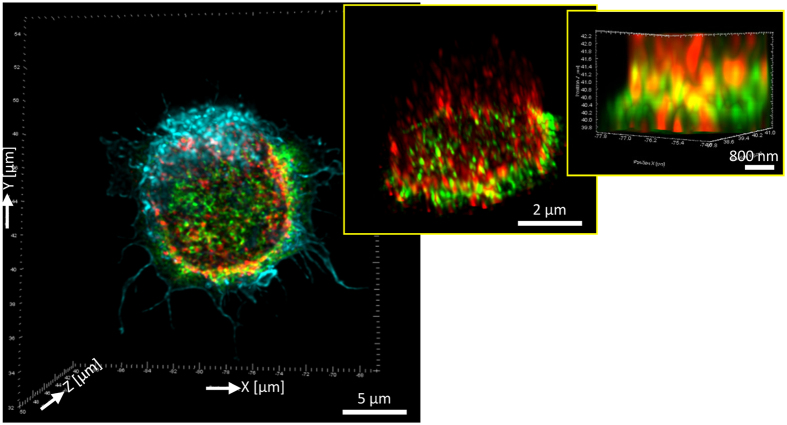
Super-resolution microscopy of GapmeR co-localization with SNX5 in human T-cells. HuT78 T-cells were incubated with 500 nM non-targeting FAM-GapmeR (green) for 6 h, fixed and counter-stained with anti-SNX5/Alexa Fluor^®^ 568 (red) and Phalloidin-Alexa Fluor^®^ 647 (light blue) and imaged by super-resolution microscopy. Insets (middle and right panels) are zoomed-in images and arrows show clear co-localization of GapmeR with SNX5.

To further validate macropinocytosis-mediated internalization of GapmeR molecules in T-cells, we immuno-stained FAM-GapmeR treated HuT78 cells for SNX5 protein (a marker of macropinosome body) and imaged using super-resolution microscopy. Co-localization of FAM-GapmeR with SNX5-positive macropinosome bodies was clearly detectable in the cytoplasm of GapmeR-treated cells (Fig. 4, Supplementary Fig. S4, Supplementary Movie 4). Of note, we couldn’t detect co-localization between GapmeR and Rab5 or Caveolin1 vesicles (data not shown). These results confirm that GapmeR cellular internalization in T-cells occurs mainly through macropinocytosis.

### Specific GapmeR molecules can effectively knockdown targeted genes of interest

**Figure 5:**
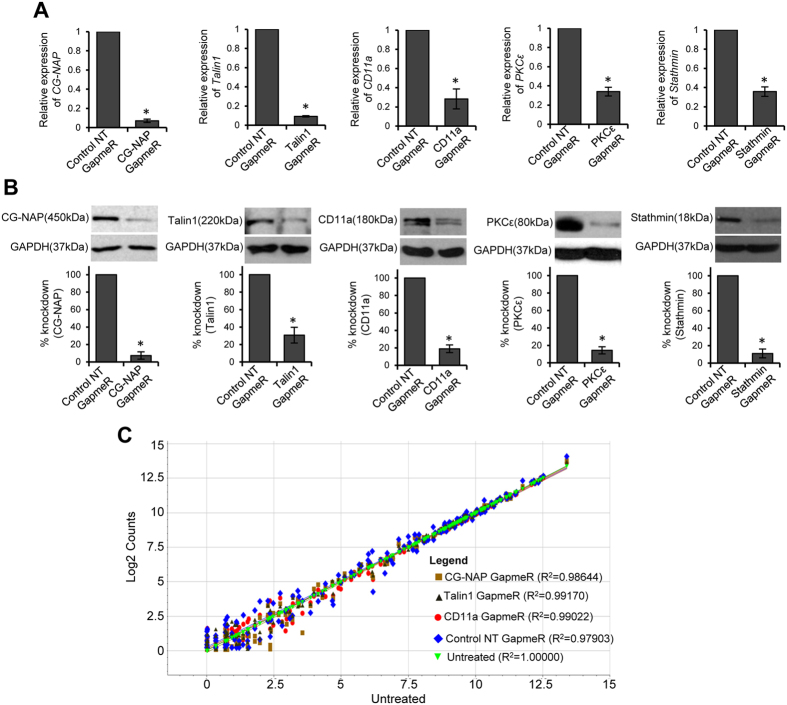
GapmeR-mediated gene silencing in human primary T-cells. Primary human T-cells were incubated separately with 500 nM GapmeR targeted against CG-NAP, Talin1, CD11a, PKCε, Stathmin or control non-targeting (*NT*) GapmeR for 48 h. (**A**) The mRNA levels of CG-NAP, Talin1, CD11a, PKCε or Stathmin were analysed by RT-qPCR. Data are fold change relative to GAPDH (mean ± SEM) of three independent experiments performed in triplicates. *p < 0.05 with respect to corresponding controls. (**B**) Cells were lysed and cellular lysates were analysed for the expression of above-mentioned proteins by Western immunoblotting. All the blots were re-probed with GAPDH as a loading control. Full-length blots are provided in the Supplementary Information file (Supplementary Fig. S11). Relative densitometric analysis of the individual protein band in terms of % knockdown is presented. Data (mean ± SEM) represent at least three independent experiments using T-cells purified from at least 3 different donors, *p < 0.05 with respect to corresponding controls. (**C**) The specificity of GapmeR-mediated knockdown in primary T-cells was determined by nanoString gene expression analysis. The scattered plot shows correlation between the expression levels of individual gene in GapmeR treated samples versus untreated control. Raw count normalized data obtained from T-cells from 2 or 3 donors is provided as Supplementary Table 1.

We next sought to determine if the macropinocytosis of GapmeR in human T-cells could be translated into effective gene silencing. For this purpose, we designed and synthesized a panel of specific GapmeR molecules targeted against 5 randomly selected genes representing a wide range of molecular weight proteins namely (i) a 450 kDa adaptor protein CG-NAP/AKAP450 (also called AKAP9), (ii) a 220 kDa cytoskeletal protein Talin1, (iii) a 180 kDa integrin receptor CD11a, (iv) a 80 kDa serine/threonine kinase PKCε isoform and (v) a highly conserved 18 kDa ubiquitous phosphoprotein Stathmin. Primary T-cells or HuT78 cells were incubated directly with the individual GapmeR molecule to allow cellular uptake for up to 48 h. After the treatment periods, the efficacy of gene silencing was determined by both RT-qPCR as well as Western immunoblotting. Macropinocytosis-mediated cellular delivery of specific GapmeR targeting the candidate proteins CG-NAP, Talin1, CD11a, PKCε or Stathmin (Table 1), effectively and significantly suppressed the expression of corresponding mRNA and proteins with specificity in primary T-cells (Fig. 5A,B, Supplementary Figs S5–S8). Moreover, the expression level of CG-NAP protein remained diminished for up to 12 days in actively proliferating T-cells that have been treated with CG-NAP GapmeR at the time of activation *via* PHA and IL-2 (Supplementary Fig. S9). These data suggest that the transient gene silencing effect of GapmeR may last for about two weeks in actively proliferating T-cells. The maintenance of a minimum dosage of GapmeR by repeated treatments may be required to suppress gene expression for longer periods of time. Notably, nucleofection of HuT78 T-cells with CG-NAP -targeting GapmeR showed comparable gene silencing that was observed using specific CG-NAP-siRNA (Supplementary Fig. S5B, left panel). Intriguingly, direct incubation (gymnotic delivery) of CG-NAP-GapmeR, but not CG-NAP-siRNA, could substantially knockdown the expression of CG-NAP protein (Supplementary Fig. S5B,C; right panels). The inability of naked siRNA delivered directly to cells to efficiently knockdown target gene could be due to modulations within the endosomes that can alter the efficiency of siRNAs by which they perform gene silencing^21^.

The specificity of targeting is an essential criteria for the application of RNAi molecules in functional gene silencing^22^,^23^. Although GapmeR design algorithm and *in silico* analysis comprehensively screen oligonucleotide sequences to avoid potential off-target effects, we performed additional experiments to rule out any potential off-target alterations in gene expressions due to GapmeR. Using nanoString nCounter gene expression analysis, we determined the expression levels of 192 different genes in human primary T-cells that were treated with 500 nM non-targeting GapmeR or GapmeR targeted against CG-NAP, Talin1 or CD11a for 48 h. We observed a strong correlation (R^2^ > 0.97) for the overall gene expression profile in T-cells treated with GapmeR molecules in comparison to that with untreated cells (Fig. 5C; raw count data given as Supplementary Table 1). Moreover, GapmeR-mediated depletion of CG-NAP or Talin-1 in primary or HuT78 T-cells showed no major effect on the expression levels of several other unrelated proteins as determined by Western immunoblot analysis (Supplementary Fig. S10). Notably, we detected reduced expression of CG-NAP protein in Talin-1 knockdown T-cells (Supplementary Fig. S10B), suggesting that CG-NAP is a potential downstream target of Talin-1 or this adaptor protein may have undergone proteolytic degradation in the absence of Talin1.

### GapmeR-mediated gene silencing identifies CG-NAP/AKAP9 and Stathmin as novel regulators of T-cell migration and chemotaxis

**Figure 6:**
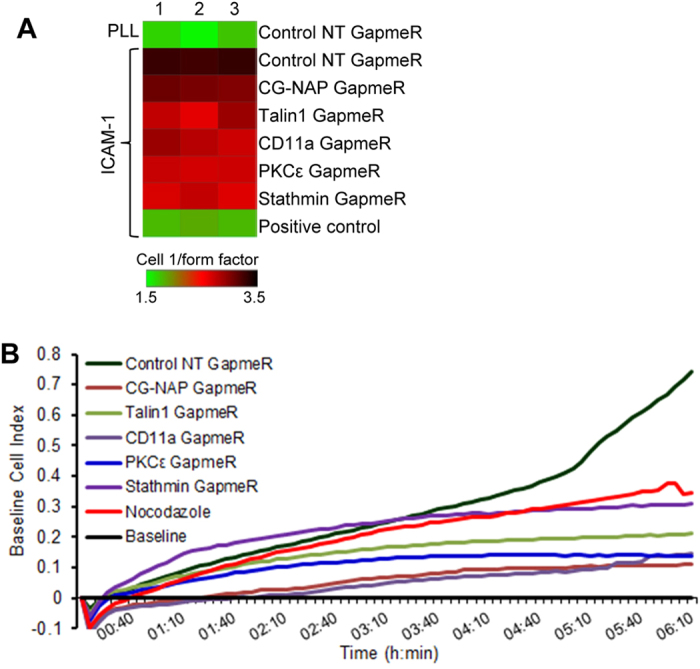
Functional effect of GapmeR-mediated gene silencing of CG-NAP, Talin1, CD11a, PKCε or Stathmin on T-cell migration. (**A**) Control or GapmeR treated primary human T-cells (2 × 10^4^ cells in 100 μl medium) were loaded in triplicates onto rICAM-1-Fc pre-coated 96-well tissue culture plates and allowed to migrate at 37 °C for 4 h. Resting T-cells were incubated on poly L-lysine (*PLL*)-coated plates and cells pre-treated with nocodazole were used as migration inhibitory control. T-cell migratory phenotypes were then automatically quantified using HCA system (cell 1/form-factor) and presented as a heatmap. (**B**) Control or GapmeR treated primary human T-cells (1 × 10^5^ cells in 100 μl medium) were loaded in triplicates onto the upper chamber of rICAM-1-Fc pre-coated CIM-Plate 16 transwell inserts. T-cell transwell migration towards SDF-1α enriched medium was automatically recorded in real-time at every 5 min interval for up to 6 h using an impedance-based detection system and quantified as “Baseline Cell Index”. Data represent at least three independent experiments using T-cells purified from at least 3 different donors.

Finally, to examine the functional influence of GapmeR-mediated gene silencing of the above 5 molecules in human primary T-cells, we performed our well-established T-cell migration assays^24^. Human primary T-cells depleted with the individual proteins (CG-NAP/AKAP9, Talin1, CD11a, PKCε or Stathmin using specific GapmeR molecules) were allowed to migrate through LFA-1/rICAM-1 cross-linking for 4 h as described previously^24^. High Content Analysis of the cells showed significant inhibition of T-cell migratory phenotypes due to the silencing of either of the above genes, including CG-NAP/AKAP9 and Stathmin (Fig. 6A). In addition, specific depletion of the selected 5 proteins individually inhibited T-cell transwell migration through ICAM-1-coated membranes towards the chemokine SDF1α, as analysed using a real-time impedance-based detection system (Fig. 6B).

## Discussion

**Figure 7:**
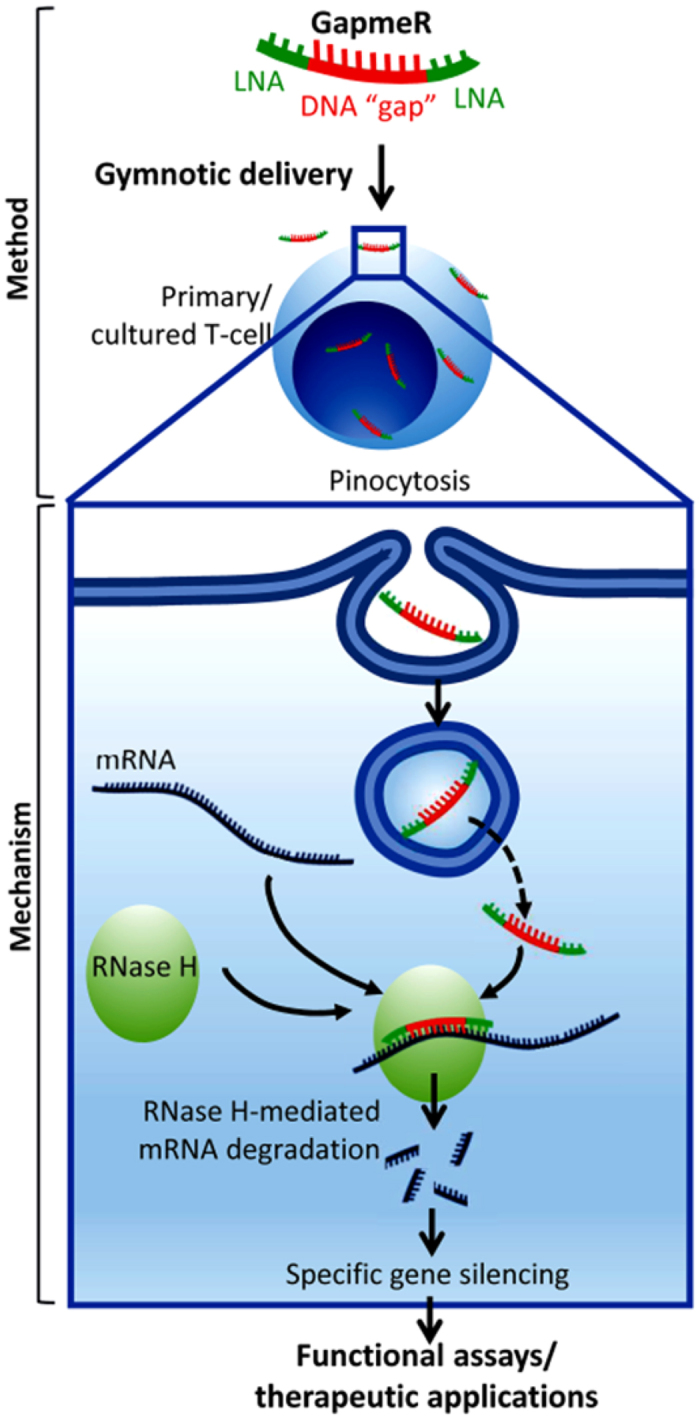
An illustration of GapmeR design, cellular internalization leading to specific gene silencing. A schematic representation of GapmeR structure, experimental work-flow for GapmeR gymnotic delivery, cellular uptake mechanism and gene silencing in human T-cells.

Efficient gene silencing in primary T-cells has been a challenge for quite some time. Primary T-cells are highly resistant to non-viral siRNA delivery or transfection using conventional RNAi methods. For example, siRNA delivery by nucleofection has limitations requiring the use of electroporation, which causes significant cell death. In addition, siRNAs show poor stability and bioavailability with low transfection efficiency, in particular, in cell types where in RNAi machinery is not efficient^4^. Thus, there is a dearth of methods to effectively maneuver the potential of gene silencing in T-cells. Herein we demonstrate that specifically designed chimeric GapmeR molecules can internalize into human primary T-cells through macropinocytosis and induce target-specific gene silencing. GapmeR-mediated gene silencing method described here is a simple and effective non-invasive approach for post-transcriptional gene silencing in human primary T-cells (Fig. 7).

In recent years, CRISPR-Cas9 genome engineering technique has emerged as a powerful tool for specific gene deletion with useful applications in the study of functional genomics, interrogation of gene functions in mammalian cells *in vitro* and in small animals *in vivo* and also for developing gene-editing therapeutics^25^. The development of a CRISPR-based toolbox to study loss/gain-of-function signaling pathways in Jurkat T-cell line has recently been reported^26^. Another study demonstrated successful knock-out of CXCR4 cell-surface receptor in human CD4^+^ T-cells with ~40% efficiency by delivering *in vitro* assembled Cas9:single-guide RNA ribonucleoproteins through electroporation^27^. The same technique was applied to generate Cas9-mediated genetic knock-in of CXCR4 and PD-1 in primary human T cells with up to ~20% efficiency^27^. However, it is to be noted that antisense GapmeR molecules knockdown targeted gene(s) at the RNA level while CRISPR-Cas9 causes knockout at the genomic DNA level in nucleus. In some cases, mammalian cells respond similarly to knockdown or knockout, but in others there may be significant differences in phenotypes that result from transient silencing of gene expression compared to complete null genotype. In this regard, on-going development in the CRISPR-Cas9 technology to modulate gene expression at the transcriptomic level would be useful. Another major challenge of CRISPR-Cas9 technology is that it requires delivery of the Cas9 gene, in addition to large size mRNA or protein, which may not be suitable for silencing gene expression in difficult to transfect cells, such as primary T-cells. In this context, self-deliverable antisense GapmeR technology described herein would be more effective in silencing gene(s) of interest in primary human T-cells.

CRISPR/Cas9 systems and RNAi molecules, such as siRNA, can cause unwanted off-target activities as reported in a number of previous studies and RNAi screens^22^,^23^,^28^,^29^. Some of these off-target effects may interfere with cell’s biochemical/metabolic pathways that can confound or complicate the interpretation of functional effects of individual gene in gene-silencing experiments and often lead to unwanted toxicities. Various strategies have been suggested to check the potential off-target effects of gene silencing, such as *in silico* prediction, gene expression analysis and rescue experiments^22^,^23^. However, these methods are only partially effective and have as-yet unproven efficacies on a genome-wide scale^23^. GapmeR molecules cause RNaseH-mediated gene silencing, independently of RNA-Induced Silencing Complex (RISC) and therefore, it does not cause RISC-associated or microRNA-like off-target activity^30^. In the present study, we employed nanoString RNA expression profiling technology platform that accurately measures the expression levels of multiple genes based on counting individual mRNA molecules with high specificity and sensitivity. Using a panel of 192 unique genes, we observed high specificity of GapmeR-mediated gene silencing with no significant off-target effects. Of note, reduced expression of CG-NAP detected in Talin-1 depleted T-cells suggests a potential role of Talin1 in the regulation or maintenance of CG-NAP expression and needs further detail investigation.

While the role of Talin1, CD11a and PKCs in T-cell migration is well-established, in this study we have further defined CG-NAP/AKAP9 and Stathmin as critical regulators of T-cell motility, which require further investigation. CG-NAP/AKAP9 has recently been shown to regulate microtubule dynamics^31^, and hence it is expected that this adaptor protein may have a crucial role in T-cell migration. Since Stathmin is a regulator of the microtubule network, it is possible that depletion of Stathmin in T-cells affected microtubule stability and dynamics ultimately resulting into reduced motility. Indeed, T-cells lacking Stathmin in Stathmin^−/−^ mice showed delayed microtubule organising centre polymerization, decreased PKCθ polarization, decreased microtubule growth rates^32^.

We believe that the GapmeR macropinocytosis is a better alternative to the conventional siRNA-based gene knockdown and can be applied to various other hard-to-transfect cell types, including naïve and effector T-cells. Use of GapmeR-mediated gene silencing will permit substantial advances in the understanding of lymphocyte biology, open-up new avenues for interactive-genomics and large-scale screening of signalling events in T-cells and contribute to the development of novel therapeutic approaches for human diseases. Specific GapmeR molecules targeted against immune checkpoint proteins coupled to the ease of delivery could potentially be used to boost the capacity of T-cells to fight against malignant diseases. In this context, our study provides exciting opportunities with regards to novel patient-friendly therapy options. GapmeR-based therapeutics could also bypass issues associated with other delivery vehicles such as toxicity and off target effects. To the best of our knowledge, this is the first report establishing GapmeR cellular internalization through macropinocytosis and GapmeR-mediated gene silencing in human T-cells. Our data positions GapmeR as a valuable tool for basic research, target screening, potential immunotherapeutic and for future gene therapy applications.

## Material and Methods

### Cell Culture

Human peripheral blood lymphocyte T-cells were expanded from buffy coat blood packs obtained from the National University Hospital Systems, Singapore as described^24^,^33^. Experiments respected institutional guidelines and were approved by the Institutional Review Board of Nanyang Technological University, Singapore. In brief, buffy coat blood (~20 ml) was diluted with an equal volume of sterile phosphate buffered saline (PBS). The diluted blood was carefully overlaid on an equal volume of Lymphoprep^TM^ (STEMCELL Singapore Pte Ltd.) in 50 ml tubes and centrifuged at 1200 × g for 20 min without brakes. Peripheral blood mononuclear cells were then removed from the liquid/Lymphoprep^TM^ interface and washed three times in PBS. Cells were then re-suspended in Gibco^TM^ RPMI 1640 medium containing 10% fetal bovine serum and antibiotics Pen Strep (all from Thermo Fisher Scientific Inc.) and incubated in Nunc^TM^ T-175 cm^2^ tissue culture flasks for 2 h in a humidified incubator at 37 °C containing 5% CO_2_. Non-adherent cells were collected and incubated in T-175 cm^2^ flasks for additional 2 h as above to remove monocytes. Finally, non-adherent cells were collected, re-suspended at 2 × 10^6^ cells/ml and stimulated with 2 μg/ml phytohemagglutinin (PHA) for 72 h at 37 °C. The cells were then washed three times and cultured with 20 ng/ml recombinant human IL-2 (Peprotech) for 5–7 days. Fresh IL-2 was added every 2–3 days and cell density was maintained between 2 to 4 × 10^6^ cells/ml. This protocol results in >98% CD3^+^ T-cells, with the proportion of T-cell subsets reported as 83% CD4^+^, 15% CD8^+^ and <1% CD4^+^/CD8^+^ cells^33^.

The human T-cell line HuT78 was obtained from the American Type Culture Collection (ATCC) and cultured as described previously^34^. Briefly, cells were cultured in Gibco^TM^ RPMI 1640 medium containing 10% fetal bovine serum, 2 mM L-glutamine and Pen Strep (all from Thermo Fisher Scientific Inc.) in a humidified chamber at 37 °C containing 5% CO_2_.

### Design and synthesis of sequence-specific GapmeR molecules and cell treatments

We designed specific GapmeR molecules against 5 selected gene targets representing a wide range of molecular weight proteins:- (i) CG-NAP, (ii) Talin1, (iii) CD11a, (iv) Protein Kinase C (PKC) ε, and (v) Stathmin using available genome-sequence database and Exiqon’s empirically developed in-house algorithm “Design tool” to achieve high target affinity, specificity, biological stability, and favourable pharmacokinetic and tissue-penetrating properties. Potential off-targets in the human transcriptomes were further examined using TagScan (http://www.isrec.isb-sib.ch/tagger), a web tool providing genome searches for short oligonucleotide sequences^35^. Antisense oligonucleotide sequences with none or least number of potential off-targets based on predicted Tm, target accessibility and no similarity with other spliced transcriptome at 0 mismatch were selected as lead GapmeR. Selected GapmeR molecules were then synthesized by Exiqon’s oligonucleotide synthesis services and used. Table 1 provides oligonucleotide sequences of GapmeR molecules targeted against 5 genes of interest, 2 each, which could effectively silence corresponding protein expression in primary human T-cells. Unlabelled or fluorescently (FAM)-labelled non-targeting GapmeR molecules (AACACGTCTATACGC, 5′ to 3′) were used as controls. Human primary T-cells or HuT78 cells (5 × 10^5^ cells in 500 μl medium) were mixed directly with various concentrations of individual GapmeR molecule (10 nM to 1000 nM, as indicated in the text and corresponding figure legends) and incubated for 6 to 72 h, depending on experimental conditions. For evaluating the effect of endocytosis inhibitors on cellular uptake of GapmeR, cells were pre-treated with various concentrations of amiloride, cytochalasin D, filipin or chlorpromazine (Sigma-Aldrich) for 30 min before adding FAM-GapmeR. In some of the experiments, cells were transfected with GapmeR or siRNA (SMARTpool, Thermo Scientific Dharmacon) through necleofection using Nucleofection^®^ Kit and 4D Nucleofector^®^ (Lonza) as per the manufacturer’s protocol.

### Cell viability assays

Cell viability was determined using CellTiter 96^®^ AQueous One solution according to the manufacturer’s instructions (Promega). The absorbance was measured at 490 nm using a microplate reader (Infinite M200 Pro, Tecan) and then percentage cell viability was calculated.

### Flow cytometry

The delivery efficiency of GapmeR into T-cells was determined using FAM-labelled non-targeting GapmeR and quantified by flow cytometry. Cells were harvested at indicated time-points and washed in PBS by centrifugation. Cells with FAM-GapmeR were analysed using BD LSR Fortessa X-20 flow cytometer equipped with BD FACSDiva™ software (BD Biosciences).

### Confocal and super-resolution microscopy

Following treatment with FAM-GapmeR, T-cells were fixed in 4% (v/v) formaldehyde. Cells were stained with Rhodamine-Phalloidin (Molecular Probes, Thermo Fisher Scientific Inc.) to visualize the cellular morphology or immuno-stained and Hoechst 33258 (Sigma-Aldrich) to visualize the nucleus. Cells were then placed on glass slides and mounted with coverslips using Fluoromount^TM^ (Sigma-Aldrich) or VECTASHIELD^®^ H-1000 (Vector Laboratories). Confocal imaging was carried out by a laser scanning microscope using a Plan-Apochromat 63X/1.40 Oil DIC objective lens and excitation wavelengths 405, 488, 561 and 640 nm (Zeiss LSM 800, Carl Zeiss). At least 20 different microscopic fields were analysed for each sample using Zen imaging software (Carl Zeiss). To determine GapmeR co-localization with specific proteins (SNX5, Rab5a and Caveolin1), FAM-GapmeR-treated HuT78 T-cells were immuno-stained with primary and corresponding labelled secondary antibodies. Cells were then imaged and processed using Leica TCS SP8 optical 3X super-resolution microscope equipped with HyVolution software (Leica). Some of the images as indicated in the relevant figure legends were acquired using wide-field or 3D-SIM on a DeltaVision OMX v4 Blaze microscope (GE Healthcare) that gives a 2-fold increase in resolution in all 3-axes (xyz) which equates to ~110 nm laterally and ~300 nm axially. The 3D-SIM technique is based upon the interaction of a structured pattern of illumination with the structures inherently present within the sample. This gives rise to Moiré fringes which are subsequently used to computationally reconstruct a super-resolved image^36^. When compared to other super-resolution microscopy techniques, 3D-SIM has the advantages of working with samples that have been prepared in a conventional way (using common fluorophores, fixation and mounting techniques) and offer 3D images of samples over a range of ~16 μm^37^. Imaris software (Andor-Bitplane, Zurich) was used to perform 3D reconstruction and to generate movies.

### ELISA assay

Primary human T-cells were incubated with 100 nM, 250 nM, or 500 nM non-targeting GapmeR for 24 h. Cells were treated with PHA as a positive control. After centrifugation, supernatant culture media were collected and secreted levels of cytokines IL-2 IL-4 IL-5 and IFN-γ were analysed by human Ready-SET-Go^®^ ELISA kits (eBioscience). The absorbance was measured at 490 nm using a microplate reader (Infinite M200 Pro, Tecan) and the amounts of secreted cytokines were calculated based on the standard curve.

### nanoString gene expression analysis

Total RNA was extracted from untreated or GapmeR treated (48 h) primary T-cells using PureLink^®^ RNA Mini Kit according to manufacturer’s instructions (Thermo Fisher Scientific). The concentration of RNA in each samples was determined using NanoDrop 2000c Spectrophotometer (Thermo Scientific). Further quality control analysis of RNA samples was performed by Agilent 2100 Bioanalyzer (Agilent Technologies). Gene expression analysis was performed using nanoString nCounter Vantage™ RNA Intracellular Signalling Panel comprising of 192 different genes, including 12 housekeeping genes as controls. A nanoString nCounter Digital Analyzer (NanoString Technologies) was used to count the digital barcodes representing the number of transcripts. The raw counts were automatically normalised by the total counts of all the tested samples and housekeeping genes in order to compensate for variations introduced by experimental procedures and counts were averaged between replicates using nSolver Analysis software, log2-transformed and presented as a scatter plot.

### Real-time quantitative PCR (RT-qPCR)

Total RNA was extracted from primary T-cells following incubation with gene specific or non-targeting GapmeR (48 h treatment) as above. A one-step RT-qPCR was performed using QuantiNova^TM^ SYBR Green RT-PCR Kit following manufacturer’s protocol (Qiagen). PrimeTime^®^ qPCR Primers for RT-qPCR assay were purchased from Integrated DNA Technologies as follows - CG-NAP, 5′CATCCGACTGAGCTTTTCTTTG3′ and 5′TTTCCTTTCTATCCCCAACCAC3′; Talin1, 5′GTTCTCCAGATCACTTTCCCC3′ and 5′CACCATCCTAACCGTCACTG3′; CD11a, 5′ACCTGGTACATGTGCTTGAC3′ and 5′GACAACTCAGCCACTACCATC3′; PKCε, 5′TACTTTGGCGATTCCTCTGG3′ and 5′CCTACCTTCTGCGATCACTG3′; and Stathmin, 5′TTCAAGACCTCAGCTTCATGG3′ and 5′AGCCCTCGGTCAAAAGAATC3′. Reactions in triplicates were analysed on a StepOnePlus^TM^ Real-Time PCR System (Applied Biosystems™) using standard mode cycle conditions: 48 °C for 30 min, 95 °C for 10 min, followed by 40 cycles at 95 °C for 15 sec and 60 °C for 1 min. Expression levels of individual mRNA (fold change) were automatically calculated against GAPDH.

### Western immunoblot analysis

At the end of the particular experiments, T-cells were washed with ice-cold PBS and lysed in the lysis buffer containing Triton X-100 (1%) and protease inhibitors as described previously^38^. The protein content of the cell lysates was determined by the Bio-Rad Protein Assay. Equal amount of cell lysates (20 μg each) were resolved on SDS-PAGE gels and subsequently transferred onto PVDF membranes. After blocking in 5% milk powder in TBS-0.05% Tween 20 (TBST) for 1 h at room temperature, the membranes were washed three times in TBST. The membranes were then incubated overnight at 4 °C with diluted primary antibodies in TBST with gentle rocking. After three washes in TBST, the membranes were incubated with HRP-conjugated secondary antibodies for 2 h at room temperature. Primary antibodies – mouse anti-CD11a (clone MEM-83, Monosan), mouse monoclonal anti-CG-NAP/AKAP9 (BD Biosciences), mouse monoclonal anti-CTLA4 (Abcam), rabbit monoclonal anti-STAT3, rabbit polyclonal anti-talin-1, rabbit anti-Stathmin and rabbit anti-GAPDH (Cell Signaling Technology). Secondary antibodies-horseradish-peroxidise-conjugated anti-rabbit and anti-mouse IgG (Cell Signaling Technology). The immunoreactive bands were visualized using the LumiGLO^®^ chemiluminescent detection system (Cell Signalling Technology) and subsequent exposure to CL-XPosure^TM^ light sensitive film (Thermo Scientific). Densitometric analyses of the Western blots were performed by using ImageJ software.

### High Content Analysis (HCA)

A High Content Analysis protocol for T-cell migration and phenotypic analysis has been optimized and established in our laboratory as described previously^24^,^34^. For T-cell migration assay, cells (2 × 10^4^ cells in 100 μl medium) pre-activated in activation buffer containing 50 mM MgCl_2_ and 15 mM EGTA to induce the high-affinity form of the LFA-1 integrin receptor were loaded in triplicates onto rICAM-1-Fc pre-coated 96-well tissue culture plates (flat bottom, Nunc™) and incubated in 5% CO_2_ at 37 °C for 4 h. Control resting T-cells were incubated on poly L-lysine (PLL) coated plates and cells pre-treated with taxol were used as positive control for migration inhibition. To quantify the amount of GapmeR in cellular cytoplasm or nuclei, cells were incubated with 500 nM FAM-GapmeR for up to 72 h. At the end of the treatments, cells were transferred into PLL pre-coated 96-well flat bottom plates. Cells were then fixed with 4% (v/v) formaldehyde in PBS, and fluorescently stained with Rhodamine-Phalloidin (Molecular Probes, Thermo Fisher Scientific Inc.) to visualize cytoplasm and Hoechst 33258 (Sigma-Aldrich) to visualize nuclei. Plates were scanned (16 randomly selected fields/well at 20X magnification) using the IN Cell Analyzer 2200 (GE Healthcare). The acquired images were automatically analysed by IN Cell Investigator software using multi-target analysis bio-application module (GE Healthcare) for nuclear and cell intensity. To quantify T-cell migratory phenotypes, cell shapes were automatically quantified into cell 1/form-factor values. A complete round shaped cells has cell 1/form-factor value = 1, whereas a polarised, migrating cell has a cell 1/form-factor value >1. The normalised values were converted into a heatmap using Spotfire© for better visualization (TIBCO Software Inc).

### T-cell transwell migration assay

Real-time transwell T-cell migration monitoring was performed at 37 °C using the CIM-Plate 16 and xCELLigence system (ACEA Biosciences). Briefly, membranes of the transwell inserts were pre-coated with 1 μg/ml rICAM-1-Fc at 4 °C overnight and blocked with 5% (w/v) BSA for 1 h at 37 °C. Untreated or GapmeR-treated (48 h treatment) human primary T-cells in triplicates were loaded in the upper chambers of CIM-Plate 16 (1 × 10^5^ cells in 100 μl medium). Cells pre-treated with taxol were used for the purpose of positive control. The upper chamber was then placed on the lower chamber of the CIM-Plate 16 containing 50 ng/ml SDF-1α enriched medium in the appropriate lower wells as an attractant. T-cells transmigrating through the membranes were automatically monitored by xCELLigence impedance-based detection system and recorded in real-time every 5 min interval for up to 6 h.

## Additional Information

**How to cite this article**: Fazil, M. H. U. T. *et al*. GapmeR cellular internalization by macropinocytosis induces sequence-specific gene silencing in human primary T-cells. *Sci. Rep.* **6**, 37721; doi: 10.1038/srep37721 (2016).

**Publisher's note:** Springer Nature remains neutral with regard to jurisdictional claims in published maps and institutional affiliations.

## Supplementary Material

Supplementary Figures

Supplementary Table 1

Supplementary Movie 1

Supplementary Movie 2

Supplementary Movie 3a

Supplementary Movie 3b

Supplementary Movie 4

## Figures and Tables

**Table 1:** The nucleotide sequence of the antisense GapmeR molecules used in the present study.

S. No.	Target proteins	GapmeR sequences (5′ to 3′)
1.	CG-NAP	*GapmeR-1:* ACTAGCCTGTAATTG
		*GapmeR-2:* GGATGCAATGCTCTTA
2.	Talin1	*GapmeR-1:* TTGGCAGTAGGATTGG
		*GapmeR-2:* CAGAGTGTCAAAGTCA
3.	CD11a	*GapmeR-1:* GATGGTAGTGGCTGAG
		*GapmeR-2:* ACGTCAATCATTAAAC
4.	PKCε	*GapmeR-1:* TAGGATGAAACTGGAA
		*GapmeR-2:* AAGCAGCAGTAGAGTT
5.	Stathmin	*GapmeR-1:* AGGTAATCAATGCAGA
		*GapmeR-2:* AGGTAATCATTGCAGA
