# Supplementary material for: GapmeR cellular internalization by macropinocytosis induces sequence-specific gene silencing in human primary T-cells
Source: Sci Rep. 2016 Nov 24;6:37721. doi: 10.1038/srep37721 (PMC5121623; doi:10.1038/srep37721)
Supplement: Supplementary Figures [file srep37721-s1.pdf]

GapmeR cellular internalization by macropinocytosis induces sequence-specific gene silencing in human primary T-cells

Mobashar Hussain Urf Turabe Fazil, Seow Theng Ong, Madhavi Latha Somaraju Chalasani, Jian Hui Low, Atish Kizhakeyil, Akshay Mamidi, Carey Fang Hui Lim, Graham D. Wright, Rajamani Lakshminarayanan, Dermot Kelleher & Navin Kumar Verma

Supplementary Data

Supplementary Movie 1. 3D projection of the confocal image shown in Figure 1C.

Supplementary Movie 2. Z-sections of the super-resolution image of a HuT78 T-cell showing internalized GapmeR molecules forming "doughnut-shaped" vesicle-like structures. Cells were treated with 500 nM FAM-GapmeR (green) and co-stained with phalloidin (cyan) and Hoechst (blue). The image was acquired by super-resolution microscopy.

Supplementary Movie 3a. 3D projection of a HuT78 T-cell showing internalized GapmeR molecules forming "doughnut-shaped" vesicle-like structures. Cells were treated with 500 nM FAM-GapmeR (green) and co-stained with phalloidin (red). The image was acquired by 3D SIM.

Supplementary Movie 3b. 3D projection of a FAM-GapmeR-treated HuT78 cell showing internalized GapmeR molecules forming "doughnut-shaped" vesicle-like structures. Cells were treated with 500 nM FAM-GapmeR (green) and co-stained with phalloidin (red) and Hoechst (blue). The image was acquired by a conventional wide-field DeltaVision OMX microscope.

Supplementary Movie 4. 3D projection of super-resolution microscopy image showing co-localization of GapmeR molecules with SNX5. HuT78 T-cells were treated with 1000 nM FAM-GapmeR (green) for 6 h, immunostained for SNX5 (red) and imaged by super-resolution microscopy.

Supplementary Figures S1 - S11.

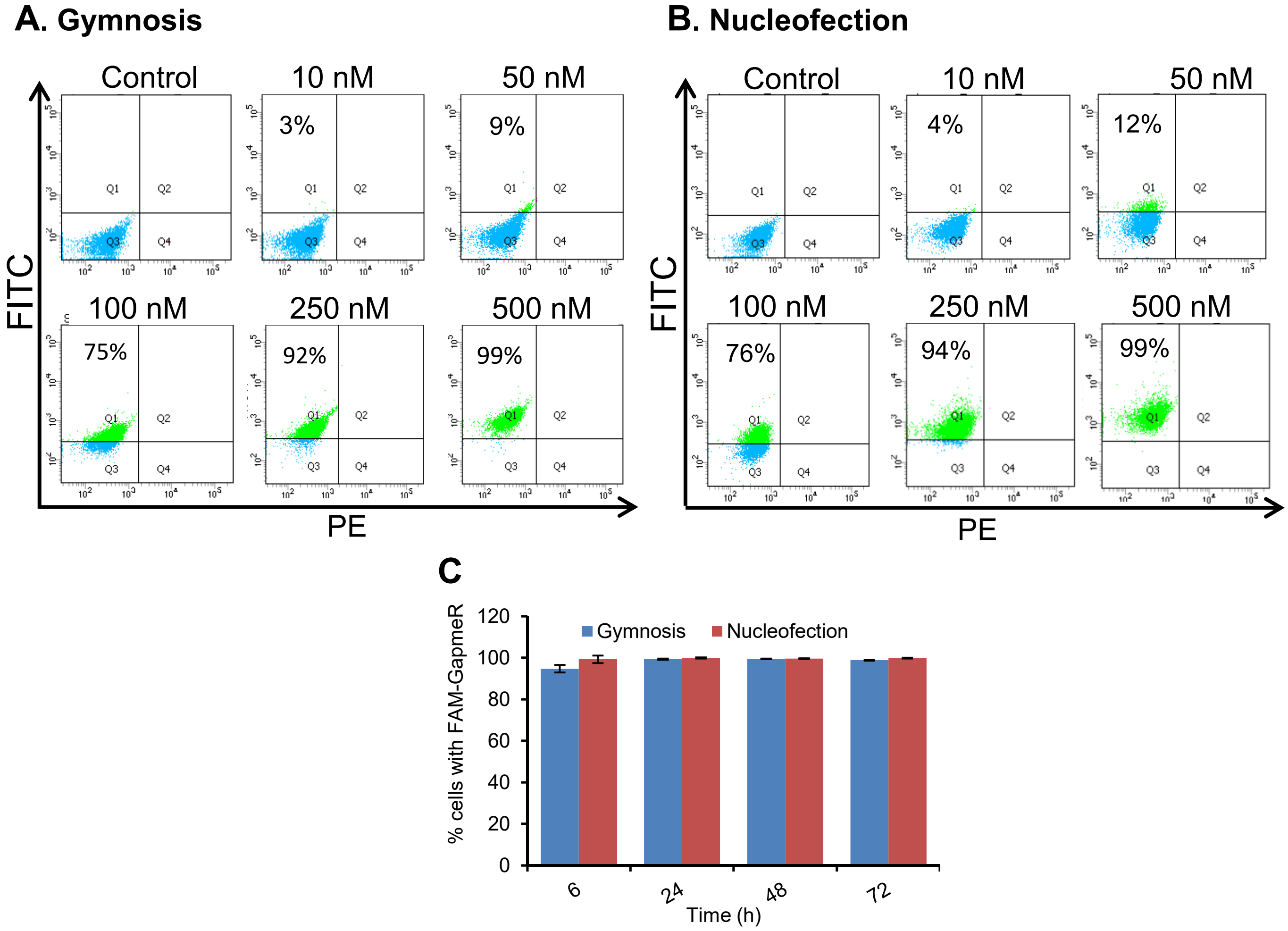

Supplementary Figure S1. Cellular internalization of GapmeR in HuT78 T-cells delivered through gymnosis or nucleofection. HuT78 cells were incubated with 10 nM, 50 nM, 100 nM, 250 nM or 500 nM non-targeting FAM-GapmeR to allow gymnosis (**A**) or transfected through nucleofection (**B**). After 48 h, GapmeR cellular uptake was analysed by flow-cytometry. Results show dose-dependent cellular internalization of GapmeR. (**C**) HuT78 cells were transfected with 500 nM FAM-GapmeR by gymnosis or nucleofection for 6 h, 24 h, 48 h or 72 h and GapmeR cellular uptake was analysed by flow cytometry. Results (mean ± SEM) show comparable cellular targeting of GapmeR delivered through either gymnosis or nucleofection. Data represent three independent experiments.

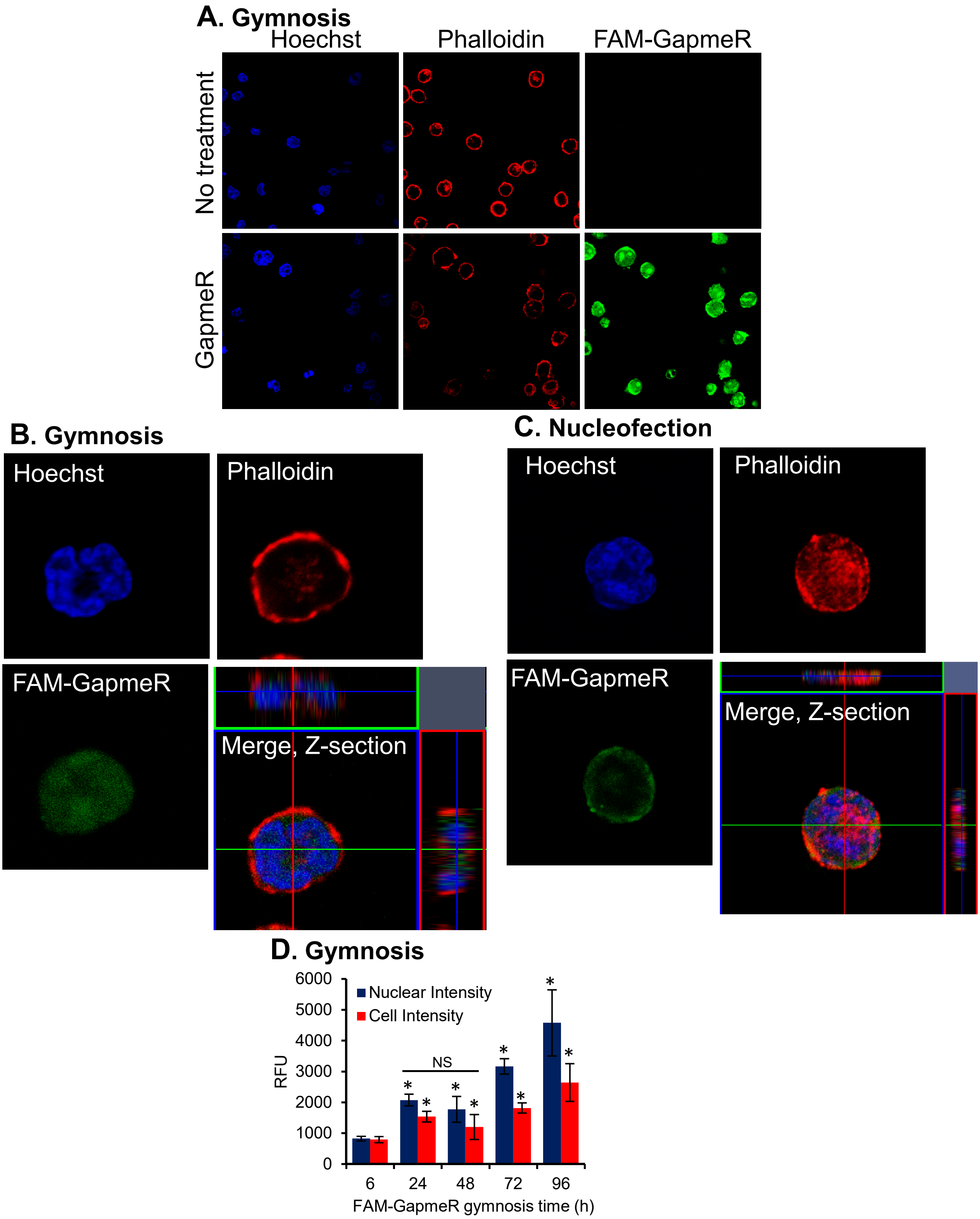

Supplementary Figure S2. Confocal microscopy of GapmeR-treated HuT78 T-cells. HuT78 cells were incubated with 500 nM non-targeting FAM-GapmeR to allow gymnosis (**A, B**) or transfected through nucleofection (**C**). After 48 h, cells were fixed and counter stained with Phalloidin-Rhodamine (to visualize cells, red) and Hoechst (to visualized nuclei, blue). GapmeR cellular localization was analysed by confocal microscopy using 63X oil objective. At least 20 microscopic fields were scanned and representative images are shown. Images clearly show cytoplasmic and nuclear localization of GapmeR. (**D**) Time-dependent cellular localization of FAM-GapmeR delivered through gymnosis was quantified by High Content Analysis (*cell intensity* and *nuclear intensity*) and presented. Data represent three independent experiments. Differences in nuclear/cell intensities between 24 h and 48 n were non-significant (NS); *p < 0.05.

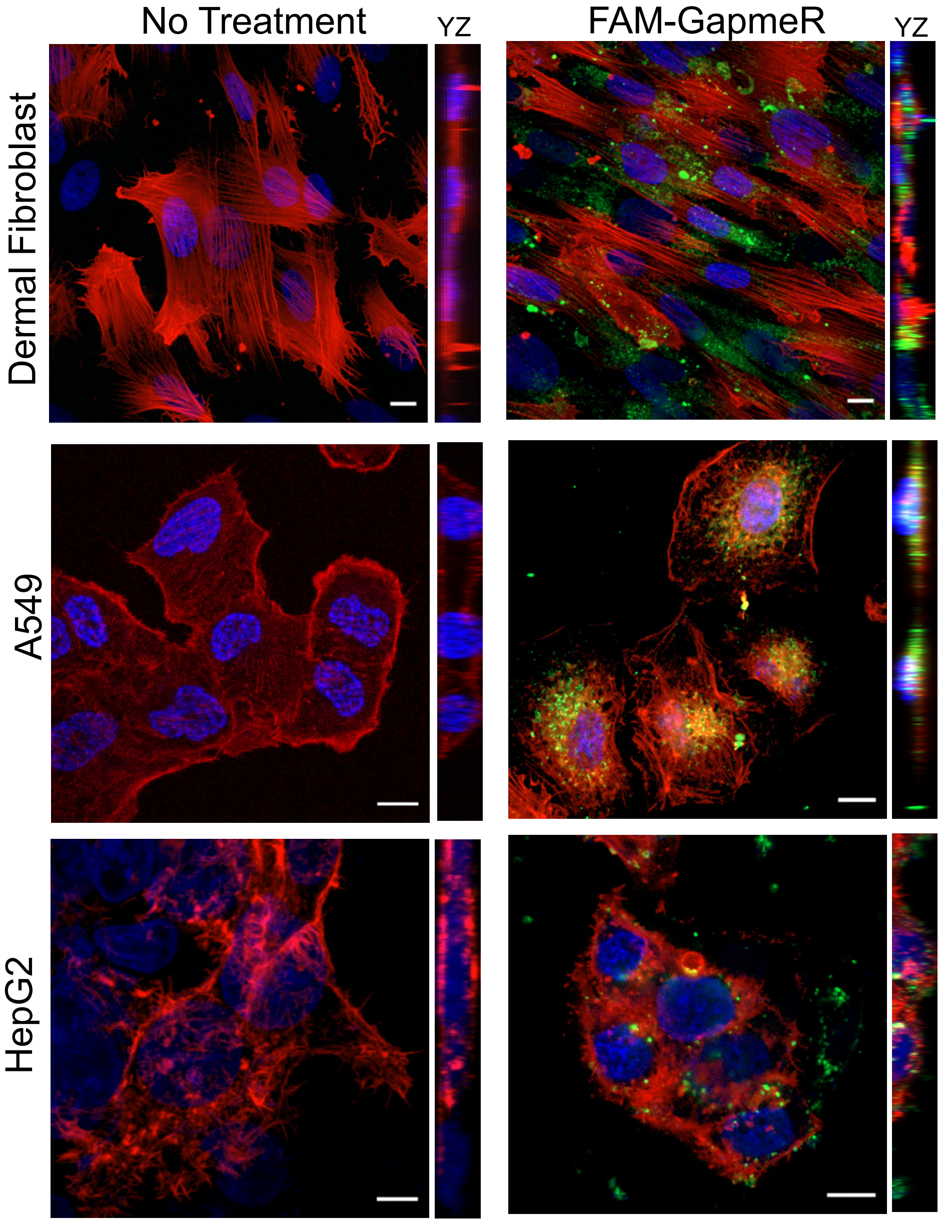

Supplementary Figure S3. Confocal microscopy of GapmeR-treated mammalian cells. Human primary dermal fibroblasts, lung epithelial carcinoma cell line A549 or hepatocellular carcinoma cell line HepG2 cells were incubated with 500 nM non-targeting FAM-GapmeR to allow gymnosis for 48 h. Cells were then fixed and counter stained with Phalloidin-Rhodamine (to visualize cells, red) and Hoechst (to visualized nuclei, blue). GapmeR cellular localization was analysed by confocal microscopy using 40X oil objective. At least 20 microscopic fields were scanned and representative images are shown. Images clearly show cytoplasmic and nuclear localization of GapmeR. Scale bar 10 µm. Z-sections (YZ) are shown besides each image.

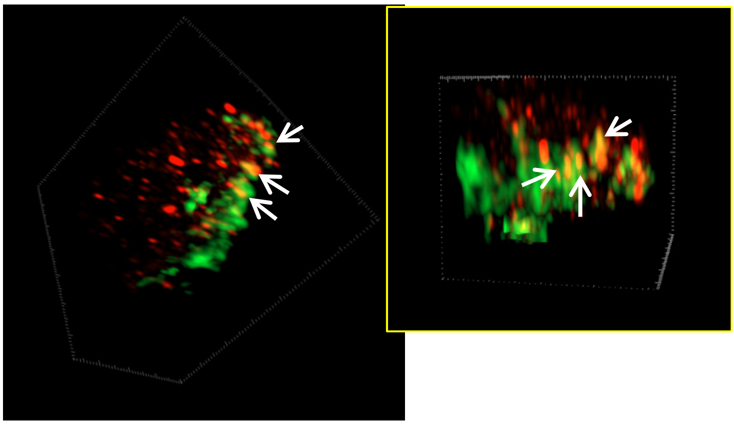

Supplementary Figure S4. Super-resolution microscopy of GapmeR co-localization with SNX5 in human T-cells. HuT78 T-cells were incubated with 1000 nM non-targeting FAM-GapmeR (green) for 6 h, fixed, counter-stained with anti-SNX5/Alexa Fluor® 568 (red) and imaged by super-resolution microscopy. Arrows show clear co-localization of GapmeR with SNX5.

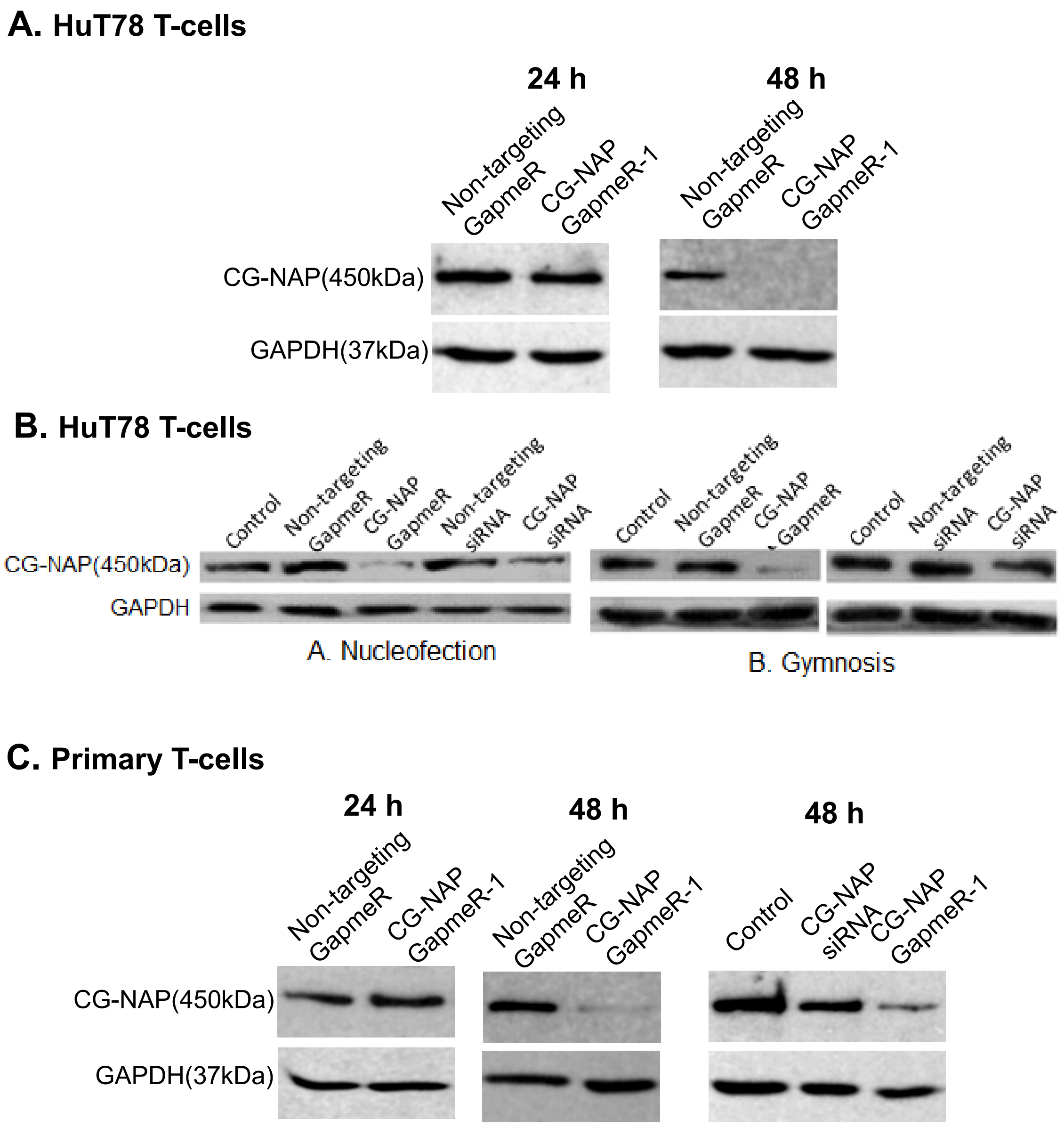

Supplementary Figure S5. Specific knockdown of a 450 kDa adaptor protein CG-NAP by targeted GapmeR in human T-cells. (**A**) HuT78 cells were incubated with 500 nM GapmeR targeted against CG-NAP (*CG-NAP GapmeR-1*) or non-targeting control to allow gymnosis for 24 or 48 h. Cells were then lysed and cellular lysates were analysed for the expression of CG-NAP by Western immunobloting. (**B**) Non-targeting or CG-NAP-targeting GapmeR or siRNA (500 nM each) was delivered to HuT78 cells through nucleofection or gymnosis. After 48 h, cells were lysed and cellular lysates were analysed for the expression of CG-NAP by Western immunobloting. (**C**) Primary human T-cells were incubated with 500 nM non-targeting control GapmeR, CG-NAP *GapmeR-1* or siRNA targeted against CG-NAP to allow gymnosis for 24 or 48 h. Cells were then lysed and cellular lysates were analysed for the expression of CG-NAP by Western immunobloting. All the blots were separately re-probed with GAPDH as a loading and specificity control. Data represent at least three independent experiments.

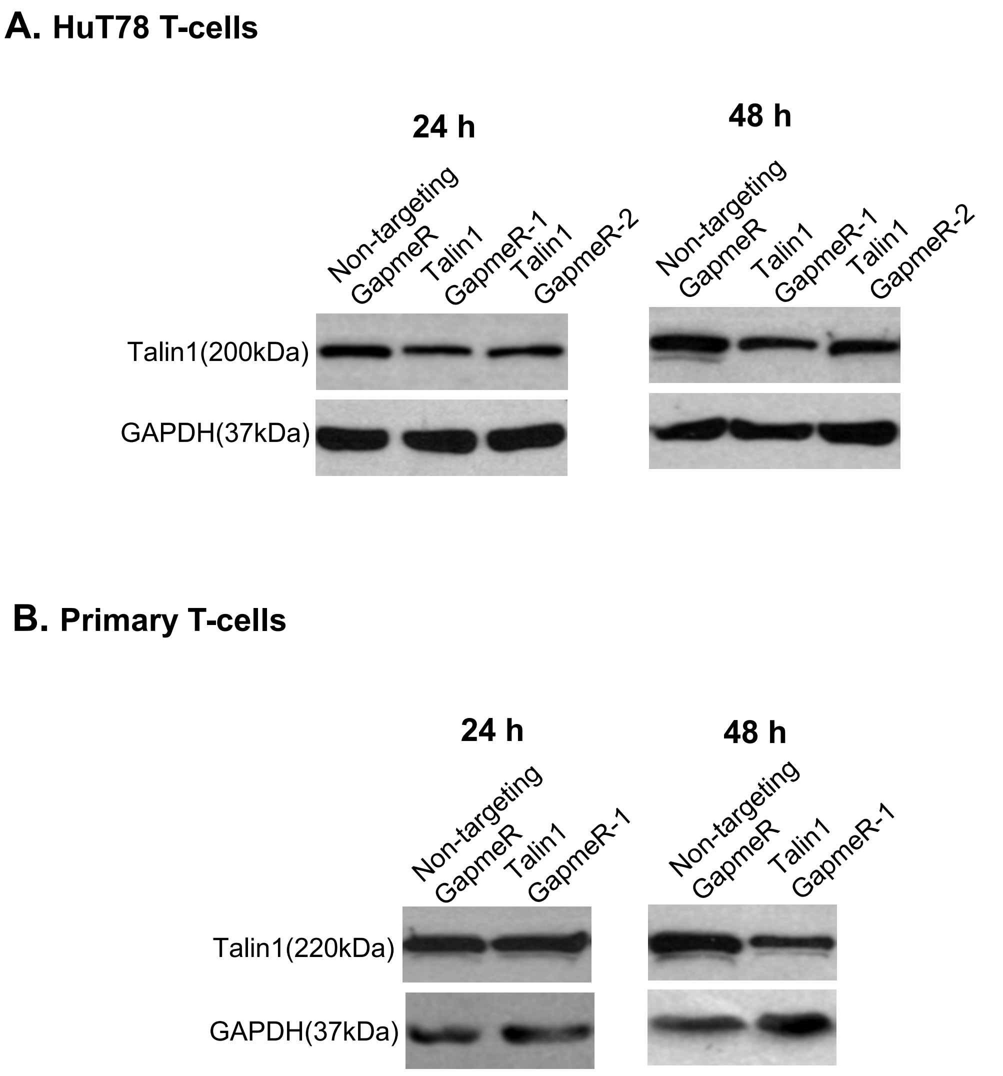

Supplementary Figure S6. Specific knockdown of a 220kDa protein Talin1 by gymnotic delivery of targeted GapmeR in human T-cells. Both HuT78 (**A**) and primary human T-cells (**B**) were incubated with 500 nM GapmeR targeted against Talin1 (2 different constructs *GapmeR-1* and *GapmeR-2* tested in HuT78 cells) or non-targeting control to allow gymnosis for 24 or 48 h. Cells were then lysed and cellular lysates were analysed for the expression of Talin1 by Western immunobloting. Blots were re-probed with GAPDH as a loading and specificity control. Data represent at least three independent experiments.

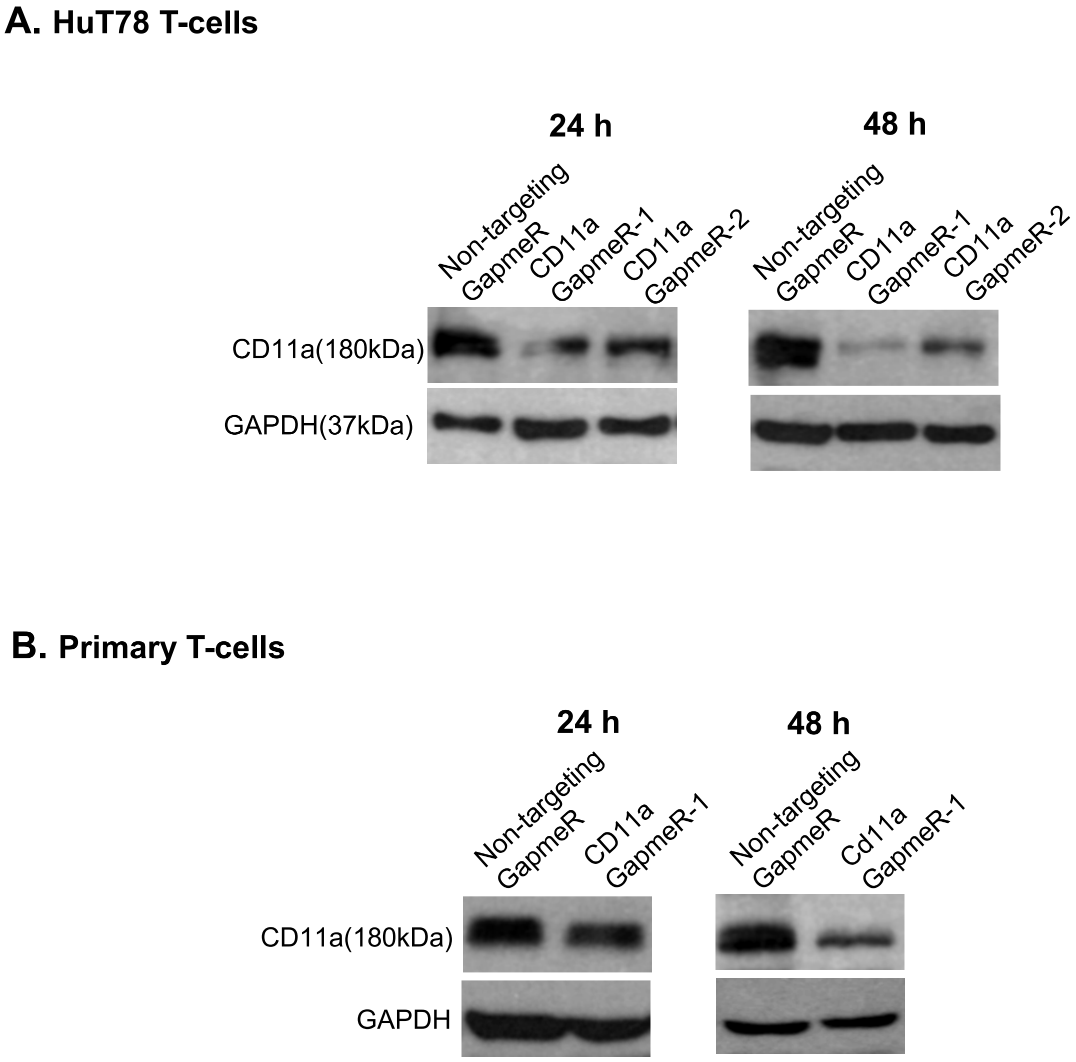

Supplementary Figure S7. Specific knockdown of a 180kDa protein CD11a by gymnotic delivery of targeted GapmeR in human T-cells. Both HuT78 (**A**) and primary human T-cells (**B**) were incubated with 500 nM GapmeR targeted against CD11a (2 different constructs *GapmeR-1* and *GapmeR-2* tested in HuT78 cells) or non-targeting control to allow gymnosis for 48 h. Cells were then lysed and cellular lysates were analysed for the expression of CD11a by Western immunobloting. Blots were re-probed with GAPDH as a loading and specificity control. Data represent at least three independent experiments.

A. **HuT78 T-cells**

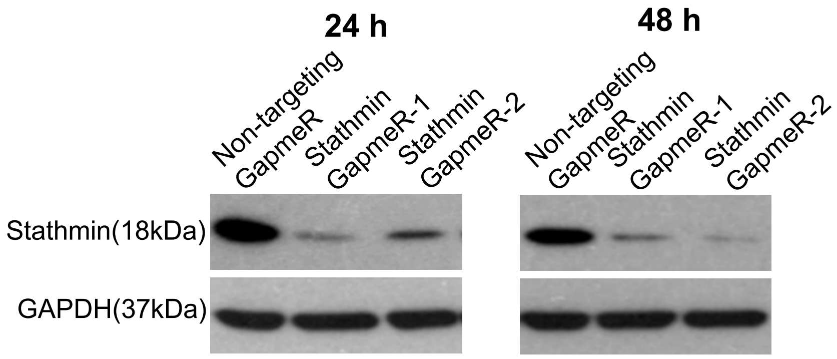

B. **Primary T-cells**

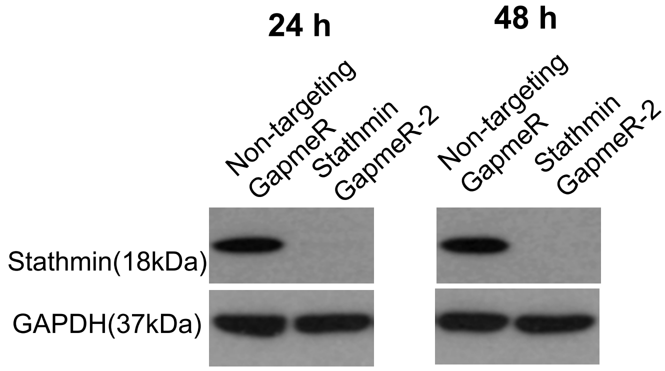

Supplementary Figure S8. Specific knockdown of a 18kDa protein stathmin by gymnotic delivery of targeted GapmeR in human T-cells. Both HuT78 (**A**) and primary human T-cells (**B**) were incubated with 500 nM GapmeR targeted against stathmin (2 different constructs *GapmeR-1* and *GapmeR-2* tested in HuT78 cells) or non-targeting control to allow gymnosis for 24 or 48 h. Cells were then lysed and cellular lysates were analysed for the expression of stathmin by Western immunobloting. Blots were re-probed with GAPDH as a loading and specificity control. Data represent at least three independent experiments.

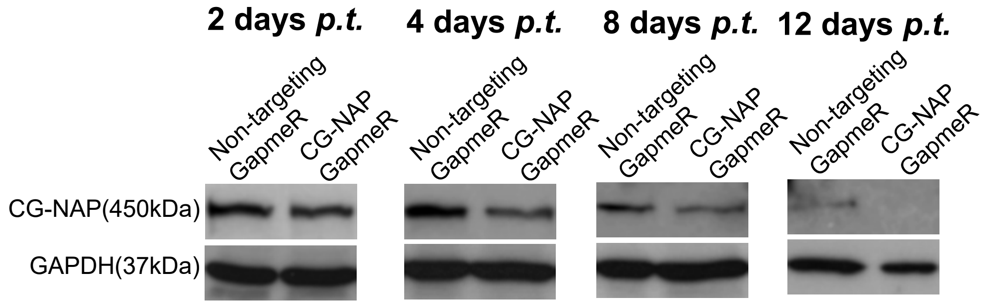

Supplementary Figure S9. Sustained knockdown of a CG-NAP by targeted GapmeR in actively proliferating primary human T-cells. Human primary T-cells were activated with PHA (day 0) and incubated with 500 nM GapmeR targeted against CG-NAP (*CG-NAP GapmeR*) or non-targeting control to allow gymnosis. Fresh medium containing IL-2 was added every 48 h. Cells were lysed at day 2, day 4, day 8 or day 12 post-treatment (*p.t.*) and cellular lysates were analysed for the expression of CG-NAP by Western immunobloting. Blots were re-probed with GAPDH as a loading control.

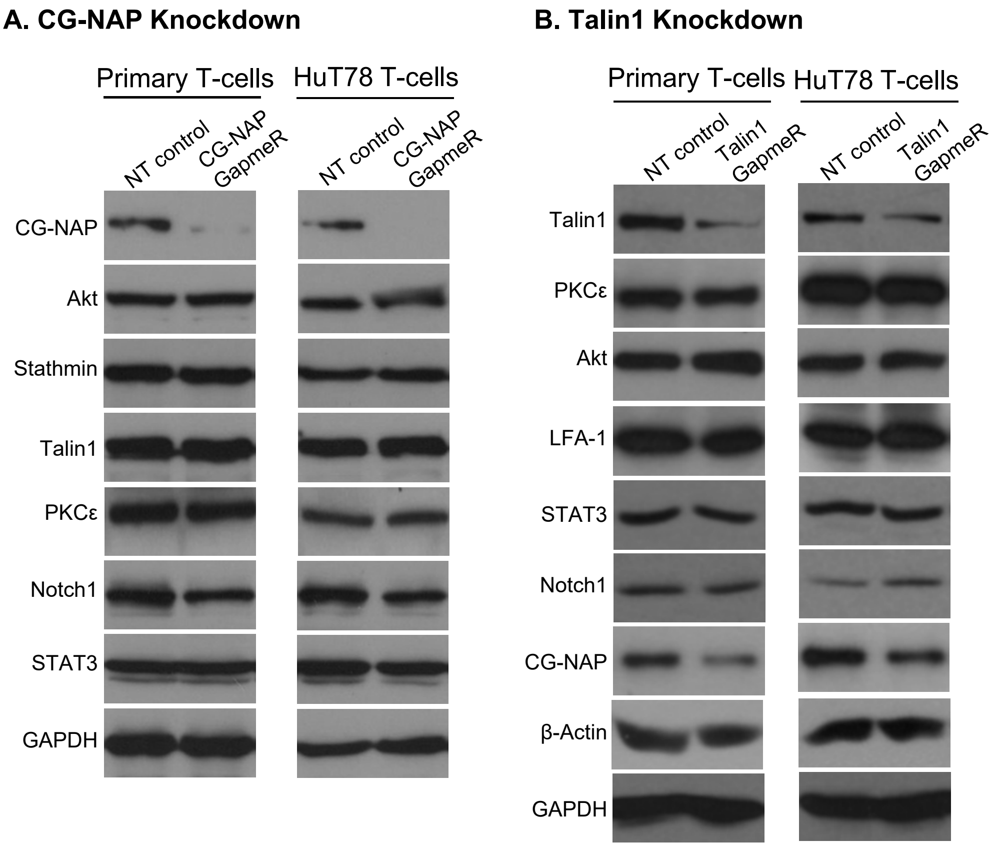

Supplementary Figure S10. Effect of GapmeR-mediated knockdown of CG-NAP or Talin1 on the expression levels of various proteins in T-cells. Primary human T-cells were incubated with 500 nM GapmeR targeted against CG-NAP (**A**) or Talin1 (**B**) to allow gymnosis for 48 h. Cells were then lysed and cellular lysates were analysed for the corresponding knockdown of CG-NAP or Talin1 by Western immunobloting. Blots were re-probed with various other proteins as indicated in the figure to determine the specificity of knockdown.

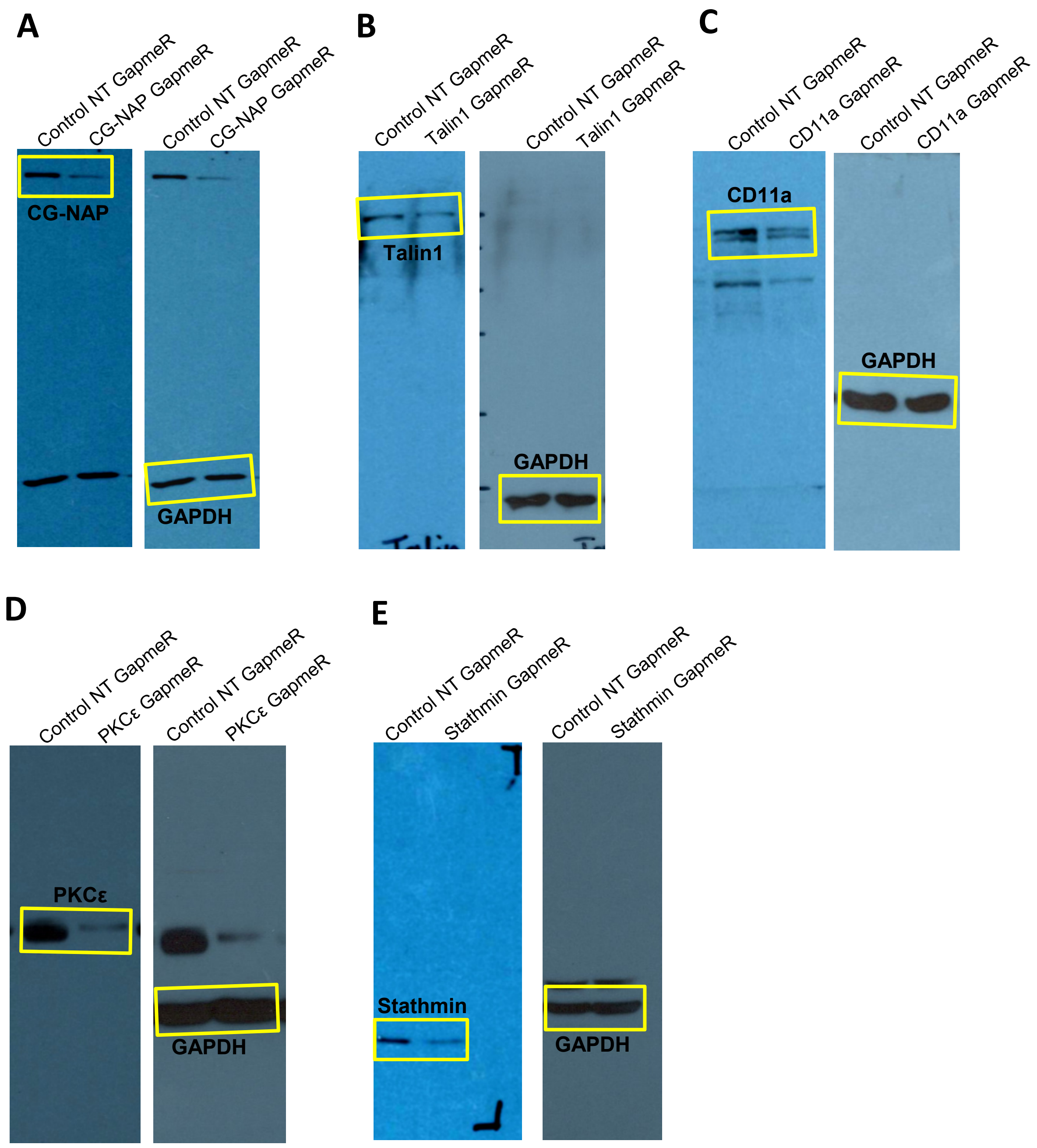

Supplementary Figure S11. Western immunoblot data showing GapmeR-mediated gene silencing in human primary T-cells that were incubated separately with 500 nM antisense GapmeR targeted against CG-NAP (**A**), Talin1 (**B**), CD11a (**C**), PKCε (**D**), Stathmin (**E**) or control non-targeting (*NT*) GapmeR for 48 h. Blots were re-probed with GAPDH as a loading control and to confirm the specificity of knockdown. The box indicates the cropped portions that were used in Figure 5 of the main article file.
